# Fabrication of In_x_Ga_1−x_N Nanowires on Tantalum Substrates by Vapor-Liquid-Solid Chemical Vapor Deposition

**DOI:** 10.3390/nano8120990

**Published:** 2018-11-29

**Authors:** Yan-Ling Hu, Yuqin Zhu, Huayu Ji, Qingyuan Luo, Ao Fu, Xin Wang, Guiyan Xu, Haobin Yang, Jiqiong Lian, Jingjing Sun, Dongya Sun, Defa Wang

**Affiliations:** 1 Fujian Provincial Key Laboratory of Functional Materials and Applications, School of Materials Science and Engineering, Xiamen University of Technology, Xiamen 361024, China; zhuyuqin1998@hotmail.com (Y.Z.); 13194071880@163.com (H.J.); 15160233004@163.com (G.X.); yhb0309001@163.com (H.Y.); 2013123202@xmut.edu.cn (J.L.); 2015000093@xmut.edu.cn (J.S.); 2013123205@xmut.edu.cn (D.S.); 2 TJU-NIMS International Collaboration Laboratory, School of Materials Science and Engineering, Tianjin University, Nankai District, Tianjin 300072, China; qyluo@tju.edu.cn (Q.L.); 15822231956@163.com (A.F.); xwangtnjrc@tju.edu.cn (X.W.); defawang@tju.edu.cn (D.W.)

**Keywords:** indium gallium nitride, gallium nitride, nanowires, chemical vapor deposition, tantalum

## Abstract

In_x_Ga_1−x_N nanowires (NWs) have drawn great attentions for their applications in optoelectronic and energy conversion devices. Compared to conventional substrates, metal substrates can offer In_x_Ga_1−x_N NW devices with better thermal conductivity, electric conductivity, and mechanic flexibility. In this article, In_x_Ga_1−x_N NWs were successfully grown on the surface of a tantalum (Ta) substrate via vapor-liquid-solid chemical vapor deposition (VLS-CVD), as characterized by X-ray diffraction (XRD), scanning electron microscope (SEM), scanning and transmission electron microscope (STEM), and photoluminescence spectroscopy (PL). It was found that the surface pretreatment of Ta and the composition of metallic catalysts played important roles in the formation of NWs. A dimpled nitrided Ta surface combined with a catalyst of nickle is suitable for VLS-CVD growth of the NWs. The obtained In_x_Ga_1−x_N NWs grew along the [1100] direction with the presence of basal stacking faults and an enriched indium composition of ~3 at.%. The successful VLS-CVD preparation of In_x_Ga_1−x_N nanowires on Ta substrates could pave the way for the large-scale manufacture of optoelectronic devices in a more cost-effective way.

## 1. Introduction

In_x_Ga_1−x_N alloys are important optoelectronic materials, which have been widely used in light emitting diodes and laser diodes due to their tunable band-gaps [1]. In_x_Ga_1−x_N alloys are also very promising for energy conversion applications such as solar water splitting [2], photocatalytic reduction of CO_2_ [3], lithium ion batteries [4], and supercapacitors [5]. In_x_Ga_1−x_N nanowires (NWs), especially, have drawn great attention due to their large surface area and improved carrier collection efficiency. A variety of In_x_Ga_1−x_N NW devices have been prepared and exhibited excellent performances [6,7,8,9,10]. Another advantage of In_x_Ga_1−x_N NWs is that they can accommodate lateral lattice mismatch with the substrate and, therefore, suppress the formation of threading dislocations. Other than conventional substrates such as sapphire, SiC, and Si, In_x_Ga_1−x_N NWs have been successfully grown on Ti [11,12], Mo [13], Ta [14], Hf [15], Ni [16], copper [17], stainless steel [18], carbon paper [4,5], etc. [19]. These new substrates have offered the In_x_Ga_1−x_N NW devices with better thermal conductivity, electric conductivity, and mechanic flexibility. Most of these growths were implemented by molecular beam epitaxy (MBE) or metallorganic chemical vapour deposition (MOCVD). Compared to MBE and MOCVD, chemical vapor deposition (CVD) is less expensive, making it more attractive to fabricate large-area devices at a lower cost.

In this article, a vapor-liquid-solid (VLS)-CVD technique was employed to grow In_x_Ga_1−x_N NWs on a pure tantalum (Ta) substrate. Ta was chosen as a substrate based on the fact that after CVD growth Ta surface would form tantalum oxides, oxynitrides, and tantalum nitrides, such as Ta_2_O_5_, TaON, Ta_3_N_5_, and Ta_x_N_y_ subnitrides. These tantalum oxides or nitrides are either conductive themselves, or have matched energy band structures with In_x_Ga_1−x_N. Ta_2_O_5_, for example, is a potential coating material, catalyst, capacitor, resistor, and optical device itself. Ta_2_O_5_ can be synthesized by thermal oxidation or electrochemically anodic oxidation [20,21]. Electrochemical anodic oxidation of Ta has been carried out to obtain a highly ordered dimpled Ta surface as a template for Au nanoparticle arrays [22], which can be used as catalyst arrays for VLS-CVD growth of nanowires. Furthermore, ammonolysis of Ta_2_O_5_ can achieve TaON and Ta_3_N_5_, both of which are promising visible-light photocatalysts [23,24]. Besides, Ta_2_O_5_, TaON, and Ta_3_N_5_ all have matched energy band structures with In_x_Ga_1−x_N [25]. Nitridation of the Ta_2_O_5_ film would also result in Ta_x_N_y_ subnitrides, which can work as conductive layers between the film and the Ta substrate [26]. Based on the above analysis, we employed both thermal oxidation and electrochemical oxidation to obtain Ta_2_O_5_ films on the surfaces of the Ta substrates. After a post-nitridation process, CVD growth of In_x_Ga_1−x_N NWs was carried out on the surface of the Ta substrates. It was found that In_x_Ga_1−x_N NWs can only be successfully fabricated on a specifically pretreated tantalum surface when Ni-Au alloys with certain compositions were used as catalysts. The microstructures of the obtained In_x_Ga_1−x_N NWs/Ta samples were extensively characterized.

## 2. Materials and Methods

Three surface states of polished Ta (99.95 %) plates were employed as substrates for the NWs growth: native, oxidative, and nitrided. Oxidative state was obtained by the heat-treatment of the polished Ta plates under atmosphere at 500 °C for 4 h. Nitrided Ta surface was achieved by a high voltage anodization followed by a high temperature nitridation. The polished Ta plates were electrochemically anodized in a two-electrode system using a platinum foil (10 × 10 mm) as the counter-electrode. The anodization was operated at 80 V in a stirred solution of concentrated H_2_SO_4_ (95–98%), H_2_O, and HF (48%) in a volume ratio of 9:4:1 for around 5–10 min until the oxide film was peeled off. The residue white films were further removed by sonication in the deionized water. The samples were then nitridated at 950 °C under 1 atm for 2 h with NH_3_ of 100 standard-state cubic centimetres per minute (sccm). 

The treated tantalum plates were then coated with mixed salts of Ni(NO_3_)_2_ and HAuCl_4_·4H_2_O as catalysts, with a volumetric percentage of the Ni(NO_3_)_2_ ethanol solution as 100%, 50%, 33.3%, 20%, and 0%, respectively. The catalysts were specified as Ni, Ni1Au1, Ni1Au2, Ni1Au4, and Au, accordingly. The treated tantalum plates with catalysts were dried at 40 °C in air before CVD growth.

**Figure 1:**
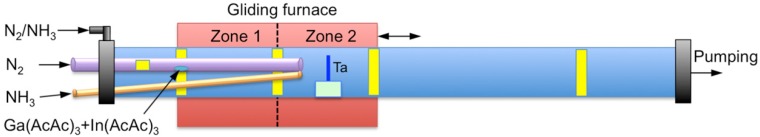
Schematic diagram of the CVD growth apparatus.

The CVD growth was carried out in a horizontal gliding furnace as shown in Figure 1 to realize fast heating and cooling. A mixture of gallium acetylacetonate (Ga(AcAc)_3_) and indium acetylacetonate (In(AcAc)_3_) (99.99%) were used as the metallic precursors to react with NH_3_ to produce In_x_Ga_1−x_N NWs [27]. The furnace has two heating zones, which were set as 400 °C and 800 °C, respectively. When the desired temperatures were attained, the furnace was glided from right to the left to align the edge of zone 1 with the metallic source, which is a mixture of 0.15 g Ga(AcAc)_3_ and 0.06 g In(AcAc)_3_. The center of zone 2 would correspond to the location of the treated Ta plate. H_2_ (20 sccm) was introduced to reduce Ni catalyst when the temperature was ramping up. Once the desired temperatures were recovered, H_2_ was turned off and N_2_ (20 sccm) was used as a carrier gas along an inner quartz tube (Φ30 mm) to transport metallic precursors to react with NH_3_ (80 sccm), which flowed through a separate inner quartz tube (Φ10 mm) to prevent parasitic reactions. The pressure was kept as 2000 Pa for 30 min. Upon the completion of the CVD growth, the furnace was glided away, and N_2_ and NH_3_ were switched to the outer quartz tube (Φ100 mm) to accelerate the cooling process.

The morphologies of the obtained samples were examined using a field emission scanning electron microscope (SEM, Zeiss Sigma 500, Netherland). The structures were identified by X-ray diffraction (XRD, Philips, PANalytical X’pert, Netherland, Cu Ka radiation (λ = 1.5417 Å)). The microstrucutre characterization of the obtained NWs was performed with Transmission Electron Microscope (TEM) using a TEM/STEM system (FEI Talos F200X, Hillsboro, OR, USA) equipped with 2 Super-X SDDs. Low temperature photoluminescence (RT-PL) was performed at 77 K with an excited wavelength of 267 nm (Horiba JobinYvon Fluorlog 3-21, USA) to evaluate the optical properties of the obtained NWs.

## 3. Results and Discussion

### 3.1. CVD Growth on the Native and Oxidative Ta Substrates

**Figure 2:**
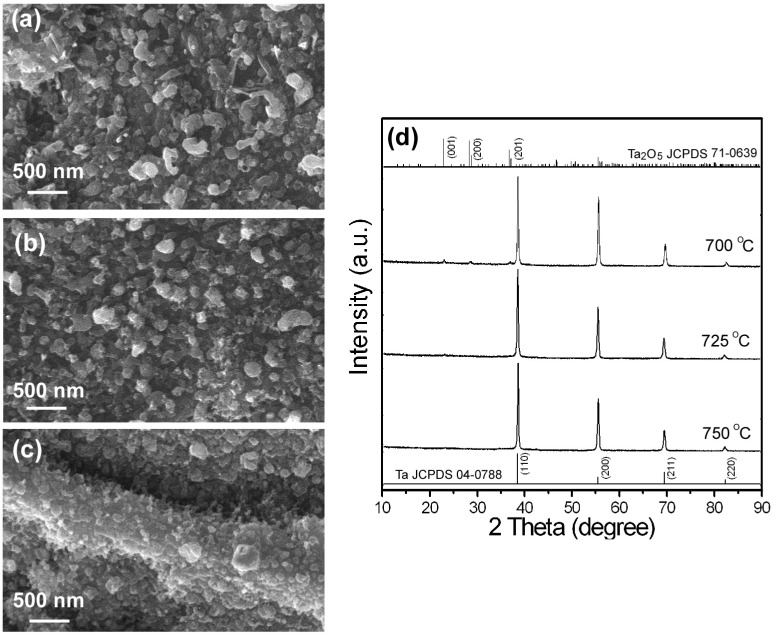
(**a**–**c**) The top-view SEM images and (**d**) the corresponding XRD spectra of the oxidative Ta samples after CVD growth using a catalyst of Ni1Au1 with the substrate temperatures of (**a**) 700 °C, (**b**) 725 °C, and (**c**) 750 °C.

After the CVD growth, neither native nor oxidative Ta surfaces can lead to the growth of NWs. On the surface of the oxidative Ta substrates, only metallic clusters, polish scratches, and film cracks can be observed in the SEM images as shown by Figure 2a–c. XRD spectra in Figure 2d showed that a crystalline β-Ta_2_O_5_ phase formed on the Ta surface, whose peak intensities decreased with increasing substrate temperatures in the order of 700 °C, 725 °C, and 750 °C. The observation indicated that the volatile nature of the Ta_2_O_5_ films could be the reason why the NWs growth failed on the native and oxidative Ta substrates. The other possibility could be originated from the reductive reaction between the Ta_2_O_5_ film and the hydrogen and ammonia during the high temperature CVD growth.

### 3.2. CVD Growth on the Nitrided Ta Substrates

**Figure 3:**
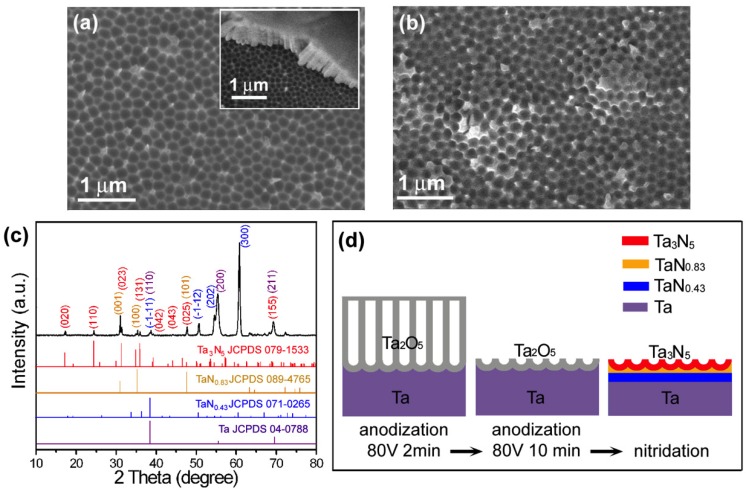
(**a**) The top-view SEM image of the Ta sample anodized at 80V for 10 min (with the inset showing the one anodized at 80V for 2 min). (**b**) The top-view SEM image and (**c**) XRD of the nitrided Ta sample. (**d**) Cross-sections schematic diagrams of the Ta surfaces.

The above results indicated that rather than an oxidative Ta surface, a nitrided Ta surface is more likely to be a substrate for the CVD grown In_x_Ga_1−x_N NWs. Nitridation of the thermally oxidative Ta substrates, however, resulted in a peeled film off the Ta substrate. As a consequence, electrochemical anodic oxidative Ta was used for the post-nitridation process. Short-time anodization of the Ta plates usually produced a thick sealed tubular oxide film with many cracks as shown by the inset of Figure 3a. On the top of the thick tubular oxide film existed a continuous passive film, which has been demonstrated to work as surface recombination centers of the electron-hole pairs and to deteriorate the device properties [28]. To solve these problems, a prolonged anodization process was used instead, which exfoliated the tubular film and exposed the underlying surface. The exposed surface contained a thin continuous oxide layer that exhibited a morphology of regular dimples ~250 nm in diameter (Figure 3a). After nitridation, the dimpled morphology was maintained and no cracks appeared on the surface (Figure 3b). XRD (Figure 3c) suggested the existence of Ta_3_N_5_, TaN_0.83_, and TaN_0.43_ on the nitrided Ta surface. The distributions of the phases were drawn schematically in Figure 3d, where it was proposed that the post-nitridation transformed the thin dimpled oxide layer into a thin dimpled Ta_3_N_5_ layer, with intermediate layers of TaN_0.83_ and TaN_0.43_ formed between the surface Ta_3_N_5_ layer and the Ta substrate.

**Figure 4:**
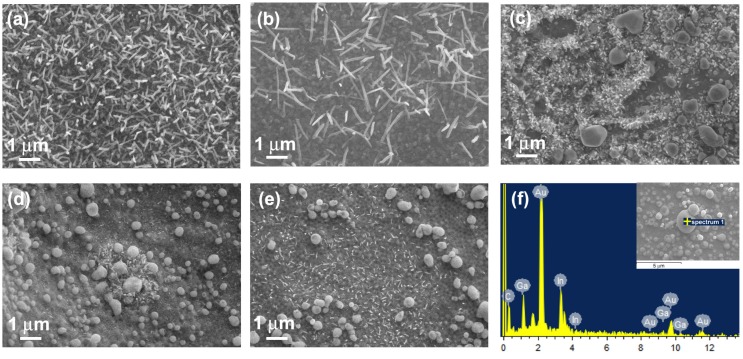
Top-view SEM images of the In_x_Ga_1−x_N NWs grown on the nitrided Ta surfaces using catalysts of (**a**) Ni, (**b**) Ni1Au1, (**c**) Ni1Au2, (**d**) Ni1Au4, and (**e**) Au. (**f**) The EDS spectrum (spectrum 1) from a large particle in the inset which is the top-view SEM image of a nitrided dimpled Ta surface after CVD growth using catalysts of Ni1Au4.

After VLS-CVD growth, homogeneous distributions of long NWs were achieved only for the catalysts of Ni and Ni1Au1 (Figure 4a–e). SEM images also show that the more the content of Au, the less the density of NWs. The large particles distributed on the surface of the nitrided Ta substrates (Figure 4c–e) were revealed to be Au particles dissolved with a large amount of Ga and In (Figure 4f). It has been reported that Au atoms can detach and migrate along GaN nanowires grown on Si during the plasma-enhanced CVD process [29]. In our case, it seems that Au atoms were dewetting on the nitrided Ta surface and coalesced to form particles, whose sizes were too large to catalyze NWs growth.

**Figure 5:**
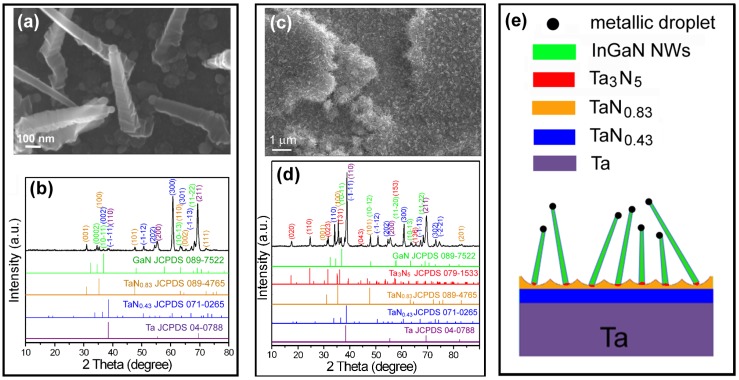
(**a**) The enlarged top-view SEM image and (**b**) XRD spectrum of the nitrided sample from a thin dimpled Ta surface; (**c**) the top-view SEM image and (**d**) XRD spectrum of the nitrided Ta sample with a thick tubular oxide film after CVD growth using catalysts of Ni1Au1. (**e**) The cross-section schematic diagram of the nitrided sample from a thin dimple Ta surface after the CVD growth.

For the catalyst of Ni1Au1, the obtained NWs had rough facets on the sidewalls and droplets on the top (Figure 5a). The later clearly indicated a VLS growth mechanism of the NWs. For the nitrided dimpled Ta surface with NWs, XRD spectrum in Figure 5b clearly revealed the presence of phases of GaN, TaN_0.83_, TaN_0.43_, and Ta, while the Ta_3_N_5_ phase was obviously missing. When a thick tubular Ta_3_N_5_ layer was used as the substrate (Figure 5c), the Ta_3_N_5_ phase remained in the XRD spectrum as shown by Figure 5d, indicating a stable Ta_3_N_5_ phase under a pressure of 2000 Pa at 800 °C. The absence of Ta_3_N_5_ peaks in Figure 5b could thereby be ascribed to the reduction of the thin dimpled Ta_3_N_5_ layer by hydrogen during the CVD growth. Although not detected by XRD, a small amount of Ta_3_N_5_ should still be present to bond the NWs and the TaN_0.83_ layer together, as shown schematically by Figure 5e. The survived Ta_3_N_5_ could be owing to the protection of the catalyst droplets. The stable Ta_3_N_5_ intermediate layer should be the main reason for the successful fabrication of nitride NWs on the Ta surface.

### 3.3. TEM Characterization of In_x_Ga_1−x_N NWs on Nitrided Ta Substrates

**Figure 6:**
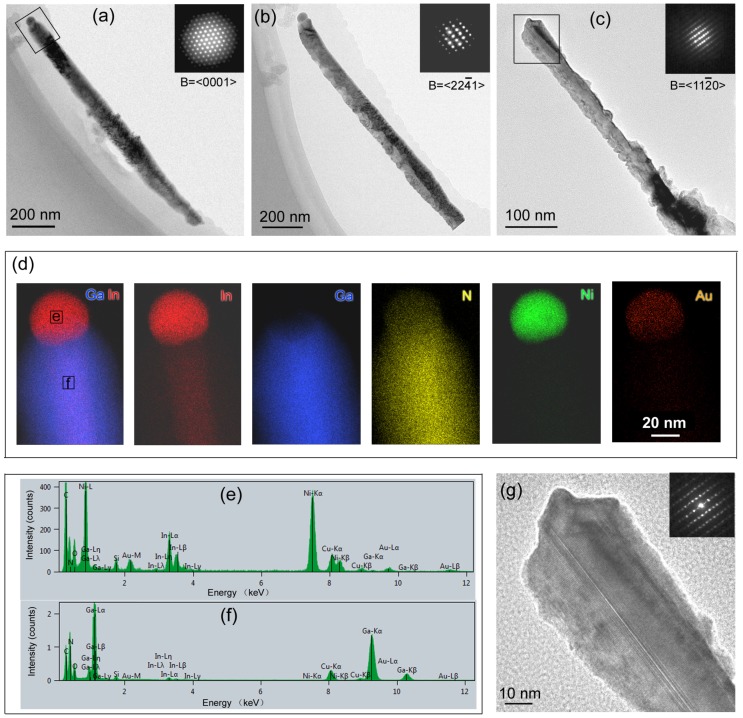
(**a**–**c**) BF TEM images and the corresponding CBED patterns (insets) of the In_x_Ga_1−x_N NWs using the Ni1Au1 as a catalyst; (**d**) STEM/EDS element mappings of the region enclosed in Figure 4a; STEM/EDS spectra of the NW from the locations (**e**,**f**) in Figure 4d. (**g**) The HRTEM image and the corresponding SADP pattern viewed along [1120] for the region enclosed in Figure 4c.

TEM characterizations were performed on the NWs, which were removed from the Ta substrate by sonication in a methanol solution and collected by the copper grids with a carbon film. Bright-field (BF) TEM images showed that the diameter of the NWs ranges from 50 to 100 nm, and their length is on the order of microns. The converged beam electron diffraction (CBED) patterns viewed along three different directions all verified a wurtzite structure with a NW growth direction of [1100] (Figure 6a–c). According to the element mappings (Figure 6d) and the EDS spectra (Figure 6e,f), neither droplets nor NWs contained Ta atoms. Instead, the droplet consisted of nitrogen and three metallic elements with a relative concentration of 75.8 at.% Ni, 22.5 at.% In, and 1.7 at.% Au (Figure 6e). The ratio of Au in the droplet was much less than the original catalyst solution (Ni1Au1), which is consistent with the SEM observation that Au atoms tend to migrate on the nitrided Ta surface. EDS/STEM mapping in Figure 6d also revealed a core-shell structure for the NW with an In_x_Ga_1−x_N core around 20–30 nm in diameter and a GaN-shell around 10–20 nm in thickness. Repeated EDS/STEM measurements were conducted on the core regions in different NWs and a typical EDS spectrum was shown in Figure 6f. Quantification results showed that the In_x_Ga_1−x_N cores had “x” varying between 1.3 to 3.5 at.%. It should be noted that since the In_x_Ga_1−x_N core was wrapped with a GaN shell, the obtained indium composition “x” is actually an average composition of the NW and could be lower than the true value of the core. High resolution TEM (HRTEM) of the NWs found no dislocations but showed the presence of the basal stacking faults (BSFs) inside the In_x_Ga_1−x_N cores (Figure 6g).

**Figure 7:**
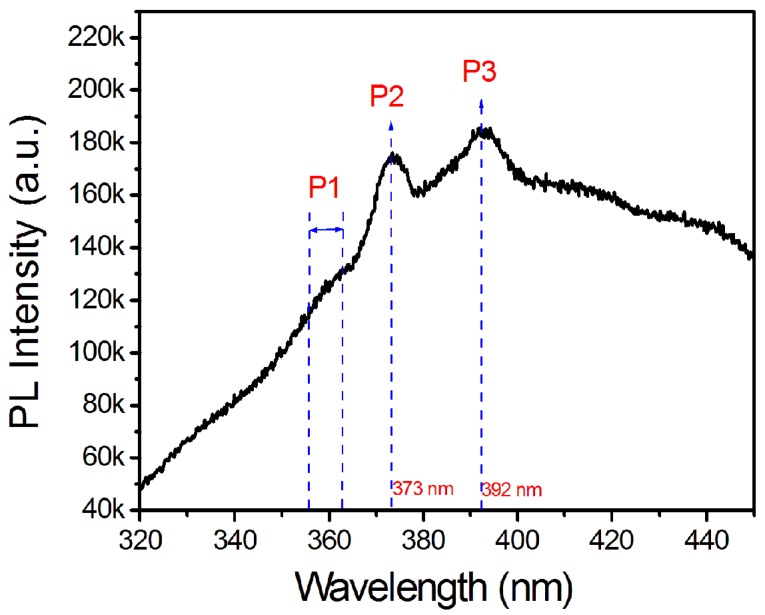
PL measurement at 77 K of In_x_Ga_1−x_N NWs on a pretreated Ta substrate using Ni as a catalyst.

Low-temperature (LT) PL was employed to characterize the NWs as shown by Figure 7. The sample showed a weak PL shoulder (P1) between 355–362 nm, which could correspond to the neutral donor bound A exciton (D0, XA) recombination (358 nm/3.472 eV) and free A exciton (XA) recombination (357.5 nm/3.477 eV) of the thin GaN shells in the NWs [14,15,30,31]. The second peak P2 is located at 373 nm, which can be attributed to the XA peak from In_x_Ga_1−x_N core. Based on P2, the band gap energy of the In_x_Ga_1−x_N cores in the NWs can be estimated as 3.32 eV. The average indium concentration *x* in the NW cores can be obtained by using equation *E_g_* = 0.7*x* + 3.477(1 − *x*) − *bx*(1 − *x*), where *E_g_*, 0.7, and 3.477 are the bandgap energy of In_x_Ga_1−x_N, InN, and GaN, respectively, and *b* represents the bowing parameter [32]. The indium concentration *x* in the NW cores can therefore be calculated to be 2.4 at.% or 3.8 at.%, depending on the choice of bowing parameter *b* as 3.6 eV [33] or 1.4 eV [32]. The indium concentration calculated from P2 peak in PL matches quite well with those measured from EDS/STEM. The third peak P3 at 392 nm could arise from two reasons. The first one is the X_A_ peak from In_x_Ga_1−x_N with higher indium composition, which is vetoed since a thorough TEM examination has been carried out and neither NW nor quantum dots with high indium concentration can be found. Therefore, it is more likely to be true to associate the relatively broad P3 peak with the structural defects such as basal stacking fault in the In_x_Ga_1−x_N core [14,15,30,31,34], which has been observed in the In_x_Ga_1−x_N core as shown in Figure 6g.

To summarize, our investigation showed that tantalum oxide is not stable under the CVD growth conditions. Instead, tantalum nitrides were demonstrated to be more suited as a substrate for In_x_Ga_1−x_N NWs. The nitrided dimpled Ta surface contained a thin continuous Ta_3_N_5_ layer, which can work as an intermediate layer to bond the In_x_Ga_1−x_N NWs and the substrate. The underlying substrate contained TaN_0.83_, TaN_0.43_, and Ta, all of which are conductive. Therefore, in terms of the transport of the photo-generated carriers, Ta should be superior to the conventional Si substrate because of an insulated Si_x_N_y_ layer formed on the surface of Si [19]. On a dimpled surface of tantalum nitrides, Au atoms tend to coalesce rather than form nano-droplets to catalyze NW growth. Ni was proved to be the suitable catalyst for the VLS-CVD growth of In_x_Ga_1−x_N NWs. The obtained core-shell structure for the NWs could be explained by an initial VLS-growth of the In_x_Ga_1−x_N core and a subsequent VS-growth of the GaN shell, which will be investigated and discussed in the future. Although not affected by the Ta substrate, the composition of In_x_Ga_1−x_N NWs should be further adjusted by changing the CVD growth parameters, such as temperature and pressure. Once higher indium content can be achieved in In_x_Ga_1−x_N NWs, a matched energy band structure can be established between the In_x_Ga_1−x_N NWs and the Ta_3_N_5_ surface layer. Furthermore, the BSFs in the m-In_x_Ga_1−x_N NWs could also help to accelerate the separation of photogenerated hole-electron pairs in the devices such as solar cells and photo-detectors. Therefore, VLS-CVD fabrication of the In_x_Ga_1−x_N NWs/Ta electrode holds great promise as a large-scale manufacture method for solar cells, photo-detectors, and photo-catalytic devices.

## 4. Conclusions

In_x_Ga_1−x_N NWs were successfully grown on Ta substrates via VLS-CVD. A Ta surface pretreatment method was developed by a combination of electrochemical anodization and high temperature nitridation, leading to a dimpled Ta_3_N_5_ surface layer with underlying intermediate layers of TaN_0.83_ and TaN_0.43_. Ni rather than Au was demonstrated as the suitable catalyst for VLS-CVD growth of NWs on the Ta templates. The obtained In_x_Ga_1−x_N NWs grew along a [1100] direction and processed an In_x_Ga_1−x_N core/GaN shell structure with the presence of basal SFs in the In_x_Ga_1−x_N core.
